# Serum calprotectin correlates with severity of severe fever with thrombocytopenia syndrome

**DOI:** 10.3389/fmicb.2025.1604243

**Published:** 2025-06-27

**Authors:** Shijie Cai, Jiahua Zhu, Zhiye Xu, Wenqin Chen, Yue Tao, Taihong Huang, Sen Wang

**Affiliations:** ^1^Department of Clinical Laboratory Medicine, Nanjing Drum Tower Hospital Clinical College of Nanjing University of Chinese Medicine, Nanjing, China; ^2^Department of Clinical Laboratory Medicine, Nanjing Drum Tower Hospital, Affiliated Hospital of Medical School, Nanjing University, Nanjing, China

**Keywords:** severe fever with thrombocytopenia syndrome, Dabie bandavirus, calprotectin, prognosis, biomarker

## Abstract

**Introduction:**

Severe fever with thrombocytopenia syndrome (SFTS) is an emerging infectious disease caused by Dabie bandavirus (DBV), clinically characterized by fever, thrombocytopenia, and multiple organ dysfunction. Severe cases are often associated with cytokine storms and exhibit a high mortality rate. Calprotectin (CP), an inflammatory marker mainly expressed in neutrophils and monocytes, has been linked to disease activity and prognosis in various inflammatory conditions. This study aimed to investigate the changes in serum calprotectin (sCP) levels and their clinical relevance in SFTS patients.

**Methods:**

Serum calprotectin levels were measured in 60 patients diagnosed with SFTS and compared with those in 60 healthy controls. The association of sCP levels with disease severity, outcome, inflammatory markers, viral load, cytokines, and clinical parameters was analyzed.

**Results:**

sCP levels were significantly elevated in SFTS patients compared to healthy controls. Severe cases and non-survivors had notably higher sCP levels than mild cases and survivors, respectively. sCP levels showed positive correlations with viral load, inflammatory cytokines (e.g., TNF-α, IL-6, IL-8, IL-10), and clinical parameters such as CRP, AST, LDH, and D-dimer. Moreover, increased sCP levels were observed in patients with renal injury, hepatic injury, and neurological symptoms.

**Discussion:**

The present study suggests that sCP levels are closely related to disease severity and prognosis, highlighting its potential as a biomarker for diagnosing and prognostic assessment in SFTS patients.

## 1 Introduction

Severe fever with thrombocytopenia syndrome (SFTS) is a disease caused by infection with Dabie bandavirus (DBV). It was first identified in China in 2009 (Yu et al., 2011) and subsequently spread widely in East and Southeast Asia (e.g., Japan, South Korea, Vietnam, Thailand, etc.) (Kim and Park, 2023). In 2019, the International Committee on Classification of Viruses officially renamed SFTS virus to DBV (Kuhn et al., 2020). DBV is a single-stranded, negatively stranded RNA virus, which is mainly transmitted by ticks (Ren et al., 2021), and domestic animals such as cattle, sheep, and dogs may also serve as reservoir hosts. The onset of SFTS is obviously seasonal, usually concentrated from March to November, with the peak season from May to July. Susceptible people are mainly located in areas where ticks are active, such as forests, hills and mountains. It is worth noting that in addition to tick-borne transmission, human-to-human transmission has been reported in some cases, and transmission routes include blood contact, droplet contact, and droplet aerosols. Therefore, research on prevention and treatment strategies for SFTS has become an important challenge in the current public health field.

The clinical manifestations of SFTS are diverse (Xu et al., 2018; Seo et al., 2021), and patients often present with fever, thrombocytopenia, enlarged lymph nodes, and gastrointestinal discomfort. Severe patients may trigger cytokine storm, severe inflammatory response syndrome and coagulation dysfunction, which in turn leads to acute multi-organ failure and high mortality. Due to the high lethality of SFTS and the potential threat to public health, early diagnosis and timely intervention are particularly important.

However, there are still many deficiencies in the diagnosis and prognostic assessment of SFTS. First, there is a lack of effective biomarkers to accurately assess the severity and prognosis of the disease. Second, although avoiding tick bites is the most direct preventive measure, early identification and accuracy of disease assessment are crucial for improving patient prognosis, as the pathogenic mechanism of SFTS has not been fully elucidated (Jin et al., 2023) and there is no effective vaccine or specific treatment available. Therefore, further research into the pathogenesis of SFTS, the search for reliable diagnostic markers, and the development of effective therapeutic treatments are current scientific issues that need to be urgently addressed.

Calprotectin (CP), also known as S100A8/S100A9 or MRP8/14, is a heterodimer consisting of S100A8 and S100A9 proteins and belongs to the S100 family of calcium-binding proteins (Korndörfer et al., 2007). CP is expressed predominantly in neutrophils and is also found in monocyte membranes. It plays an important role in inflammation and immune responses, especially in neutrophil-mediated inflammation. CP exerts antibacterial effects by competing with bacteria for zinc ions (Zygiel and Nolan, 2018), and also participates in the recruitment of inflammatory cells and endothelial cell interactions, which further exacerbate the inflammatory response.

CP has been extensively studied in a variety of inflammatory and autoimmune diseases. For example, in inflammatory bowel disease (IBD), fecal CP (Fecal Calprotectin, fCP) has been shown to be a reliable biomarker for assessing disease activity and predicting relapse. In addition, serum calprotectin (sCP) can also be used as a marker for some inflammatory diseases. sCP shows potential to correlate with disease activity in autoimmune diseases such as rheumatoid arthritis (RA) and systemic lupus erythematosus (SLE). Particularly in COVID-19, sCP levels were significantly correlated with disease severity and adverse clinical outcomes such as mechanical ventilation and multi-organ failure.

However, there is a gap in the current research on the changes of sCP during the course of SFTS and its correlation mechanism with disease severity. In this study, we investigated the correlation between the level of sCP and various clinical parameters in order to analyze its changes during the course of SFTS and its clinical significance.

## 2 Materials and methods

### 2.1 Patients and control participants

This study analyzed a total of 60 hospitalized patients diagnosed with fever and thrombocytopenia from April 2024 to July 2024 in Nanjing Drum Tower Hospital, China. This study was approved by the Ethical Review Board (IRB) of Nanjing Drum Tower Hospital, China (2022-238-02). All patients were tested for DBV RNA using real-time reverse transcription polymerase chain reaction to confirm DBV infection. The patient cohort consisted of 28 males and 32 females with a mean age of (65.4 ± 9.9) years. SFTS patients were categorized into two groups based on their prognosis: survivor and non-survivor groups. Severe cases were defined as patients who met any of the following criteria (Deng et al., 2013): multiple organ dysfunction, acute respiratory distress syndrome (ARDS), sepsis, disseminated intravascular coagulation (DIC), failure of one or more organs (e.g., heart failure, acute renal failure, or liver failure), infection-induced toxic shock, or death. Sixty subjects were recruited from the Nanjing Drum Tower Hospital Physical Examination Center as a control group. In the healthy control group, there were 32 males and 28 females with a mean age of (66.1 ± 13.5) years. No significant differences in gender or age were observed between the control and patient groups.

### 2.2 Detection and collection of clinical parameters

In this study, a variety of technical equipment was used for the detection of clinical indicators: quantitative serum ferritin testing was performed on a fully automated chemiluminescence immunoassay platform (Siemens Atellica IM1600); inflammation and liver function indicators (CRP, ALT, AST) and metabolism-related enzyme profiles (LDH, etc.) were determined using a Beckman biochemical analyzers; hematology parameters (complete blood count, platelet dynamics, hemoglobin level) were obtained by Sysmex XN series blood analysis system; coagulation function assessment (PT, APTT, FIB, D-Dimer) was done by Sysmex CS-5100 fully automated coagulation analysis system.

For virological testing, serum DBV RNA enrichment was accomplished based on a fully automated nucleic acid extraction system by the magnetic bead method in conjunction with a special purification kit, and absolute quantification of viral load was performed by fluorescence quantitative PCR technology (ABI 7500 system) in combination with an DBV-specific primer probe kit. Cytokine profiling (IL-1β, IL-2, IL-4, IL-5, IL-6, IL-8, IL-10, IL-12p70, IL-17, IFN-γ, IFN-α, TNF-α) was performed by applying flow cytometry technology (EasyCell platform, Hangzhou Bohao Technology), and standardized operation procedures were strictly followed. All experimental data were collected double-blind through hospital LIS system and EMR system to ensure data traceability.

### 2.3 Measurement of sCP by ELISA

sCP levels were quantitatively measured using ELISA (Buhlmann Laboratories AG). All serum samples used for sCP measurement were collected on the day of hospital admission, to ensure consistency across all enrolled patients. All reagents were equilibrated to room temperature prior to use. Serum samples were diluted 1:50 with incubation buffer and allowed to stand for 15 min. Standards, quality controls, and diluted samples (100 μL per well, in duplicate) were added to the ELISA plate. The plate was sealed and incubated on a horizontal shaker at 800–1000 rpm for 30 min. After washing three times, 100 μL of enzyme conjugate was added to each well and incubated for another 30 min. Plates were then washed five times, and 100 μL of TMB substrate solution was added per well and incubated in the dark for 15 min. The reaction was stopped, and absorbance was measured at 450 nm. sCP concentrations were calculated based on the standard curve.

### 2.4 Receiver operating characteristic (ROC) curve analysis

ROC curve analysis was performed to evaluate the prognostic utility of sCP levels in SFTS patients, including distinguishing severe from mild cases and predicting mortality. Optimal cut-off values were determined using the Youden index (sensitivity + specificity − 1), and predictive performance was quantified by the area under the ROC curve (AUC).

### 2.5 Statistical analysis

Student’s *t*-test was used to compare the differences between SFTS patients and healthy controls, and the differences were considered statistically significant when *p* < 0.05. All data measurements were expressed as mean and standard deviation m ± S. Pearson correlation analysis was used to study the correlation between clinical parameters. A significant correlation was considered when the r value was > 0.2 and *p* < 0.05. Statistical analysis and plotting were performed using GraphPad Prism 8.3 software.

## 3 Results

### 3.1 Baseline characteristics and clinical profiles of patients

This study included 60 patients diagnosed with SFTS, comprising 48 survivors (80%) and 12 non-survivors (20%). Based on disease severity, patients were classified into mild cases (*n* = 34, 57%) and severe cases (*n* = 26, 43%). Detailed baseline characteristics and clinical parameters of all patients are presented in Table 1. Our analysis indicated that non-survivors tended to be older compared to survivors. Additionally, non-survivors had a significantly higher prevalence of underlying cerebrovascular, renal, and hepatic diseases compared with survivors; however, there were no notable differences between the two groups in terms of hypertension or diabetes prevalence. Laboratory analyses revealed significant differences in ALT, AST, and LDH levels between survivors and non-survivors, as well as between severe and mild cases.

**Table 1:** Baseline characteristics for patients with SFTS.

Parameters	Survival	Non-survival	*p*	Mild symptoms	Severe symptoms	*p*
No.	48	12	–	34	26	–
Male/Female (*n*)	21/27	7/5	–	14/20	14/12	–
Age (years)	63.77 ± 9.19	71.3 ± 12.84	0.0238	62.0 ± 9.1	69.5 ± 10.5	0.0046
Days of hospital stay	7.27 ± 6.37	4.83 ± 1.95	0.20	6.12 ± 4.20	7.65 ± 7.45	0.32
Time from onset to admission (days)	4.96 ± 2.13	5.17 ± 3.56	0.79	4.94 ± 2.07	5.08 ± 2.91	0.83
History *n* (%)						
Hypertension	16 (33%)	3 (25%)	0.58	11 (32%)	8 (31%)	0.90
Diabetes	8 (17%)	5 (42%)	0.06	5 (15%)	5 (19%)	0.64
Cardiovascular disease	24 (50%)	6 (50%)	1.00	15 (44%)	15 (58%)	0.30
Cerebrovascular disease	4 (8%)	8 (67%)	<0.0001	1 (3%)	11 (42%)	0.0002
Kidney disease	4 (8%)	7 (58%)	<0.0001	0 (0%)	11 (42%)	<0.0001
Liver disease	32 (67%)	12 (100%)	0.02	21 (62%)	23 (88%)	0.02
Cancer	0 (0%)	1 (8%)	0.04	0 (0%)	1 (4%)	0.25
Laboratory findings						
WBC (×10^9^/L)	3.5 ± 2.3	3.6 ± 1.7	0.94	3.5 ± 2.8	3.6 ± 2.2	0.84
NEU (×10^9^/L)	2.4 ± 2.0	2.3 ± 1.4	0.86	2.4 ± 2.0	2.4 ± 1.8	0.88
LYM (×10^9^/L)	0.9 ± 0.6	1.0 ± 0.7	0.49	0.8 ± 0.6	1.0 ± 0.7	0.47
HGB (g/L)	132.1 ± 18.0	139.3 ± 20.0	0.23	131.8 ± 18.0	135.7 ± 20.0	0.43
PLT (×10^9^/L)	58.9 ± 24.3	46.9 ± 23.0	0.13	60.9 ± 23.1	50.8 ± 25.0	0.11
ALT (U/L)	70.61 ± 54.5	272.7 ± 213.6	<0.0001	60.4 ± 32.7	180.3 ± 180.7	0.0006
AST (U/L)	196.8 ± 183.7	747.4 ± 662.6	<0.0001	147.5 ± 127.4	511.7 ± 522.5	0.0003
LDH (U/L)	928.2 ± 1,046	2,162 ± 1790	0.003	677.2 ± 738.8	1807 ± 1,597	0.0008
ALB (g/L)	35.9 ± 6.5	34.4 ± 6.1	0.48	37.4 ± 6.0	33.4 ± 6.3	0.02
GLB (g/L)	30.2 ± 5.1	32.8 ± 3.5	0.11	30.6 ± 4.7	31.0 ± 5.3	0.75
CRP (mg/L)	4.9 ± 5.5	5.9 ± 4.0	0.6	4.7 ± 6.4	5.6 ± 3.7	0.5
IL-6 (pg/ml)	26.8 ± 49.1	35.9 ± 27.0	0.66	17.8 ± 16.5	44.8 ± 55.3	0.08

The data was presented as the mean ± standard deviation (SD). BMI, Body Mass Index; WBC, White Blood Cell; NEU, Neutrophil; LYM, Lymphocyte; HGB, Hemoglobin; PLT, Platelets; ALT, Alanine Aminotransferase; AST, Aspartate Aminotransferase; LDH, Lactate Dehydrogenase; ALB, Albumin; GLB, Globulin; CRP, C-Reactive Protein.

### 3.2 SFTS patients exhibited significantly higher sCP levels compared to healthy controls

**Figure 1:**
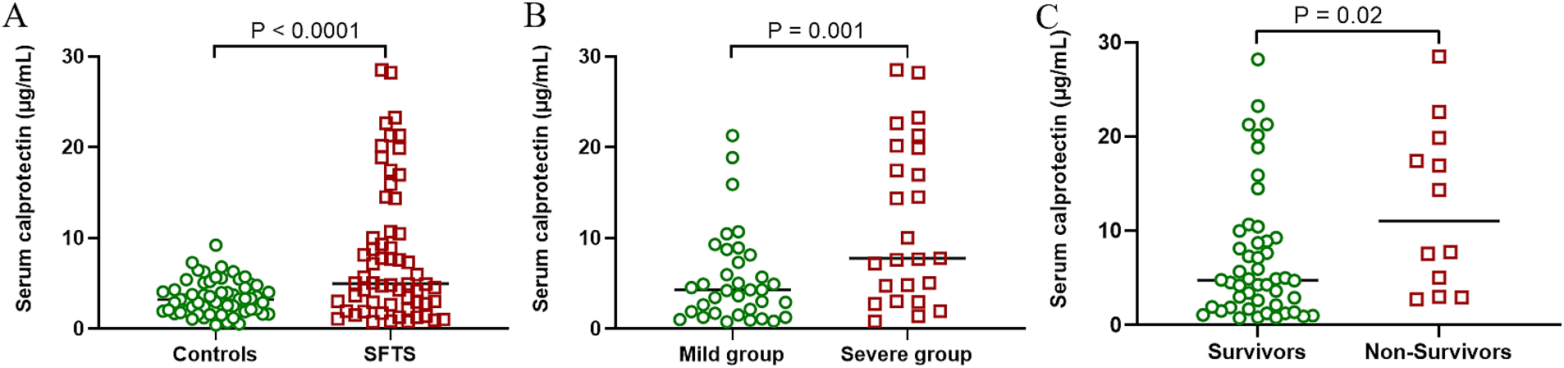
SFTS patients had significantly higher sCP levels than healthy controls. Comparison of sCP between healthy individuals and SFTS patients **(A)**. Comparison of sCP between patients with mild SFTS and patients with severe SFTS **(B)**. Comparison of sCP levels between survivors and non-survivors **(C)**.

To investigate alterations in sCP levels among SFTS patients, this study compared and analyzed sCP levels between SFTS patients and healthy controls. The results showed that SFTS patients had significantly higher sCP levels compared to healthy controls (*p* < 0.0001) (Figure 1A), suggesting a potential association between sCP and the inflammatory response in SFTS. Further analysis based on disease severity revealed significantly higher sCP levels in severe patients compared with mild patients (*p* = 0.001) (Figure 1B), indicating that elevated sCP levels might reflect disease severity. Additionally, when comparing sCP levels between non-survivors and survivors, the results demonstrated significantly higher sCP levels in non-survivors (*p* = 0.02) (Figure 1C). ROC curve analysis showed that sCP had moderate predictive performance in distinguishing severe from mild cases (AUC = 0.71) and in predicting mortality (AUC = 0.70) (Supplementary Figure S1).

### 3.3 sCP levels significantly correlated with viral load and platelet count

**Figure 2:**
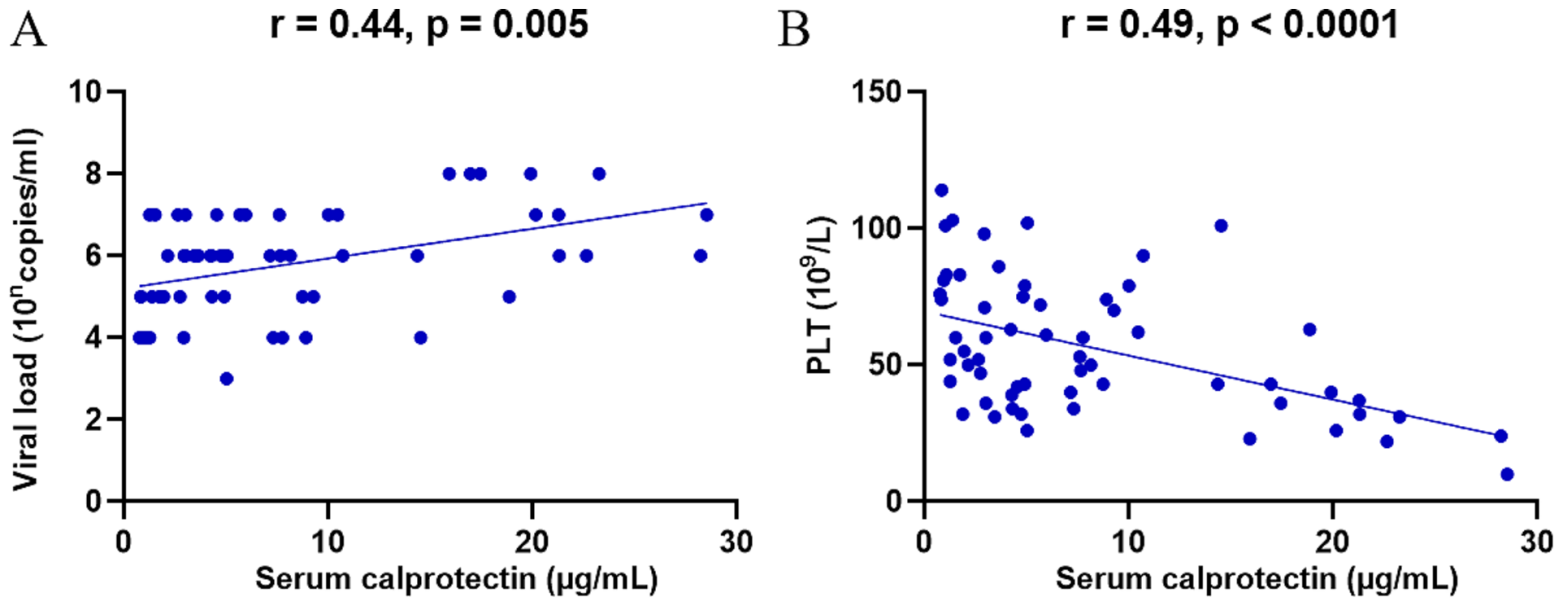
sCP was significantly correlated with viral load and platelet count. sCP levels in SFTS patients were analyzed for correlation with viral load and platelet count, respectively **(A,B)**.

SFTS is a disease caused by DBV infection, and the viral load reflects disease severity to some extent. To further explore the role of sCP in SFTS, we analyzed the relationship between sCP levels and serum viral load. The results revealed a significant positive correlation between sCP levels and viral load (*r* = 0.44, *p* = 0.005) (Figure 2A), suggesting that elevated sCP levels may reflect increased viral replication. Additionally, decreased platelet count is a characteristic clinical manifestation of SFTS and serves as an indicator of disease severity. We further assessed the correlation between sCP levels and platelet counts, which showed a significant negative correlation (*r* = −0.49, *p* < 0.0001) (Figure 2B).

### 3.4 Correlation between sCP levels and peripheral blood cell counts

**Figure 3:**
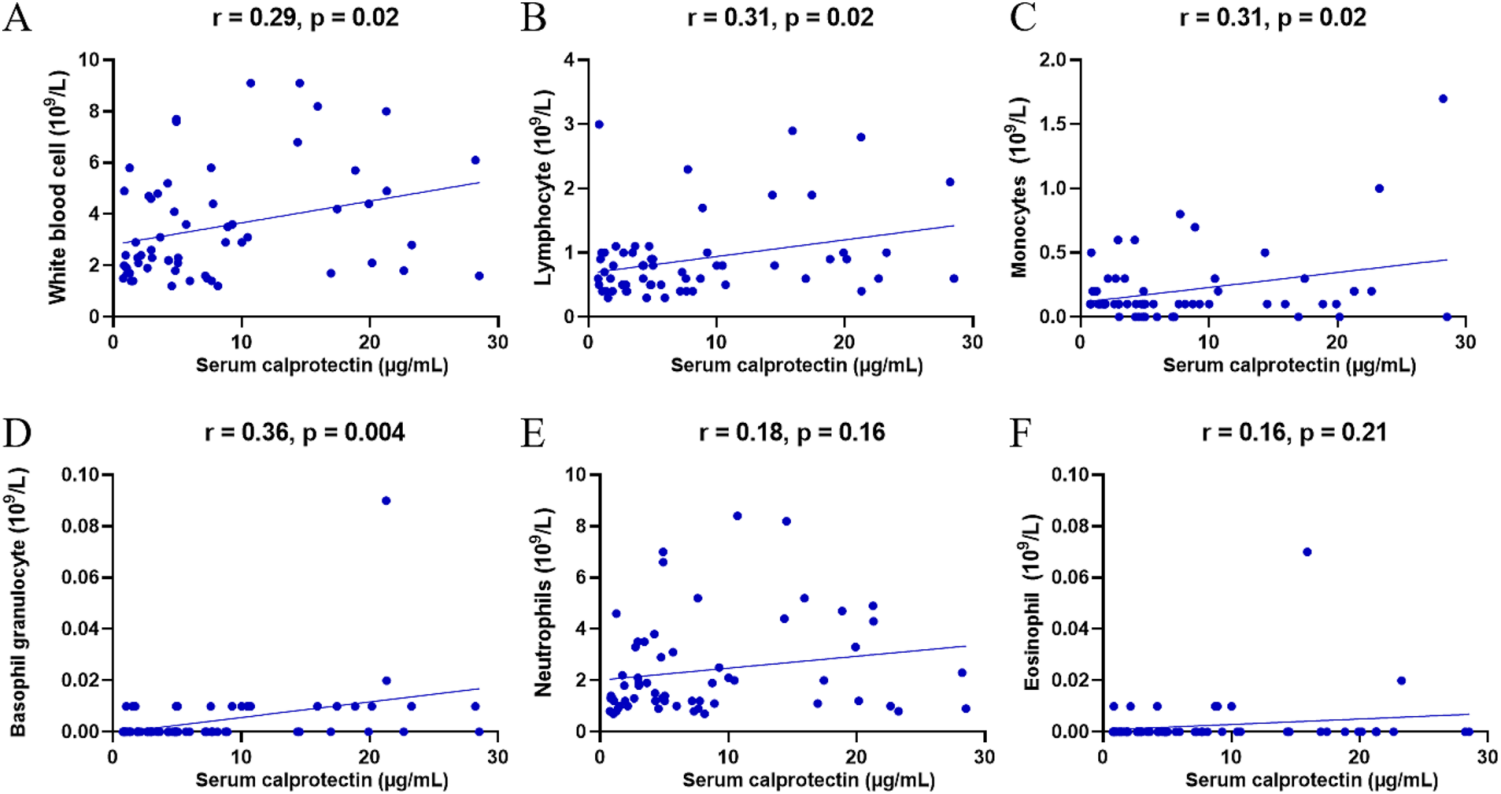
Correlation between sCP and peripheral blood cells. sCP levels in SFTS patients were analyzed for correlation with leukocytes, lymphocytes, monocytes, basophils, neutrophils, and eosinophils, respectively **(A–F)**.

Previous studies indicated that neutrophils and monocytes/macrophages are the primary sources of sCP. To further elucidate the association between sCP and immune cells during the progression of SFTS, this study analyzed correlations between sCP levels and various peripheral blood cell counts. The results demonstrated significant positive correlations between sCP levels and counts of total leukocytes (*r* = 0.29, *p* = 0.02) (Figure 3A), lymphocytes (*r* = 0.31, *p* = 0.02) (Figure 3B), monocytes (*r* = 0.31, *p* = 0.02) (Figure 3C), and basophils (*r* = 0.36, *p* = 0.004) (Figure 3D), while no significant correlations were observed with neutrophils (r = 0.18, *p* = 0.16) or eosinophils (r = 0.16, p = 0.21) (Figures 3E,F). These findings provide valuable insights for further understanding the immunopathological role of sCP in SFTS.

### 3.5 Correlation between sCP levels and serum cytokine profiles

**Figure 4:**
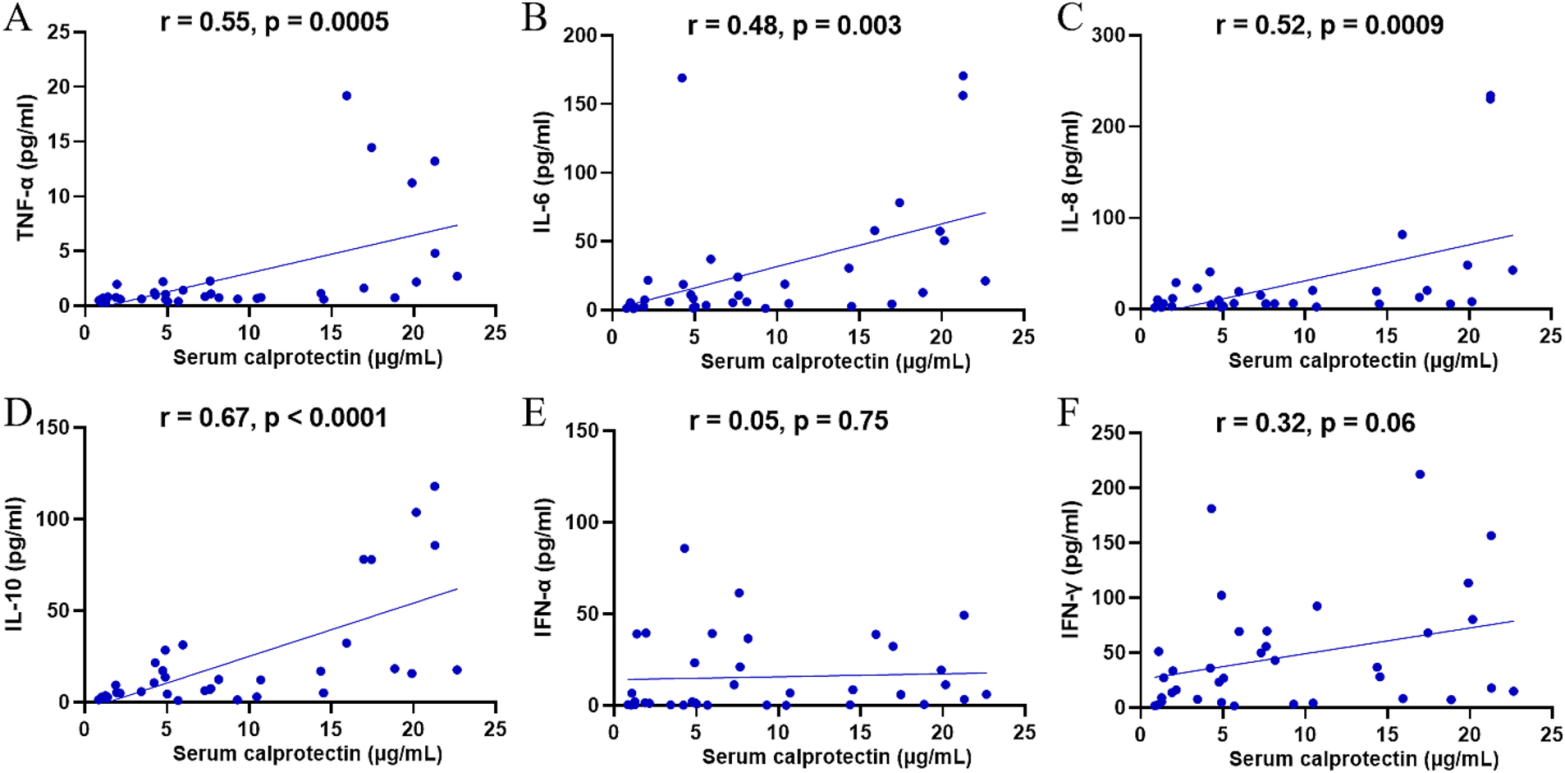
Correlation of sCP with serum cytokines. sCP levels in SFTS patients were analyzed for correlation with TNF-α, IL-6, IL-8, IL-10, IFN-α, and IFN-γ, respectively **(A–F)**.

An excessive inflammatory response may trigger a cytokine storm, causing severe clinical manifestations in SFTS patients. Previous studies have demonstrated significantly elevated serum cytokine levels in patients with SFTS. To further elucidate the relationship between sCP and inflammatory responses in SFTS patients, we examined the correlations between sCP levels and various serum cytokines. The results showed significant positive correlations between sCP levels and serum concentrations of TNF-α (*r* = 0.55, *p* = 0.0005) (Figure 4A), IL-6 (*r* = 0.48, *p* = 0.003) (Figure 4B), IL-8 (*r* = 0.52, *p* = 0.0009) (Figure 4C), and IL-10 (*r* = 0.67, *p* < 0.0001) (Figure 4D). These findings suggest that sCP may contribute to regulating the inflammatory response and is closely associated with cytokine release in SFTS patients. However, no significant correlations were observed between sCP levels and either IFN-α (*r* = 0.05, *p* = 0.75) (Figure 4E) or IFN-*γ* (*r* = 0.32, *p* = 0.06) (Figure 4F).

### 3.6 Clinical relevance of sCP in relation to laboratory parameters

**Figure 5:**
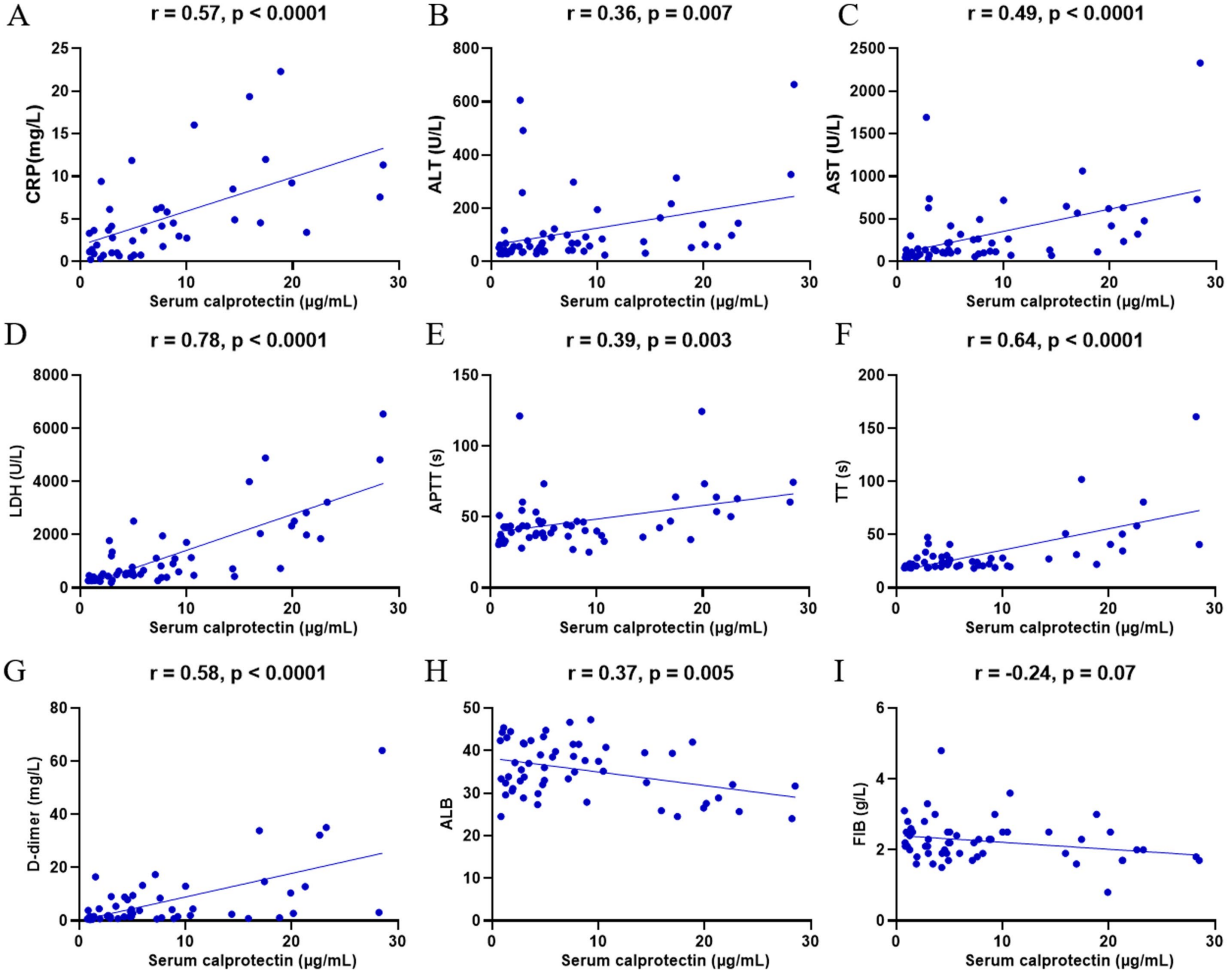
Correlation of sCP with clinical parameters. sCP levels in SFTS patients were analyzed for correlation with CRP, ALT, AST, LDH, APTT, TT, D-dimer, FIB, and ALB, respectively **(A–I)**.

DBV infection induces significant alterations in various clinical laboratory parameters, reflecting disease severity. To elucidate the clinical relevance of sCP in SFTS, we analyzed its correlations with selected clinical indicators. The results demonstrated that sCP levels were significantly positively correlated with CRP, ALT, AST, LDH, APTT, TT, and D-dimer (Figures 5A–G), and negatively correlated with ALB (Figure 5H). However, no significant correlations were observed between sCP and FIB, PCT, HDL, LDL, PT, or GLB (Figure 5I; Supplementary Figure S2).

### 3.7 Association of sCP levels with clinical manifestations and complications in patients with SFTS

**Figure 6:**
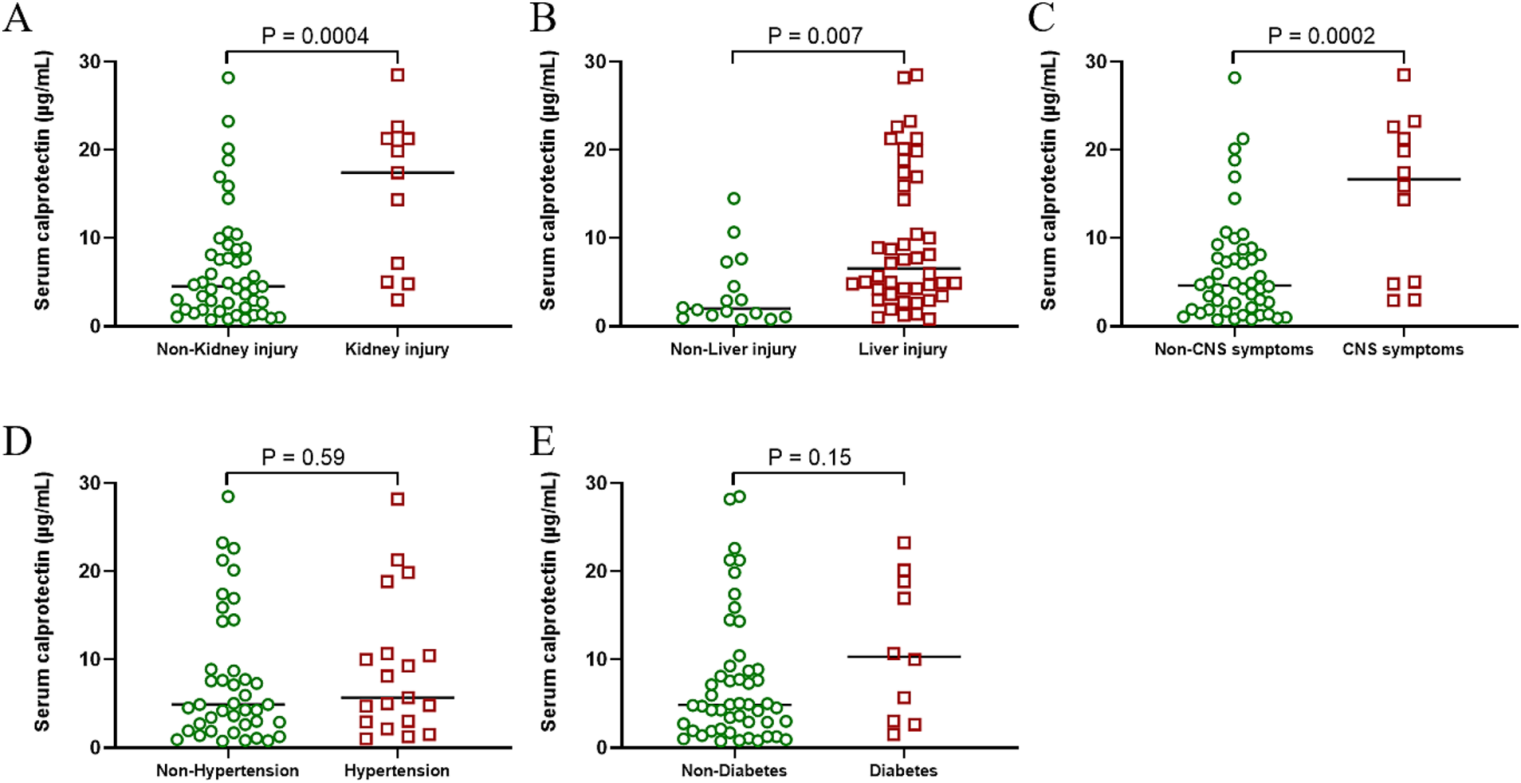
Correlation between sCP and patient complications. Comparison of sCP between SFTS patients with or without renal injury, hepatic injury, neurological symptoms, hypertension, and diabetic symptoms **(A–E)**.

Patients with severe SFTS often present with neurological impairment, pronounced inflammatory responses, and acute multiple-organ failure—clinical features commonly associated with elevated mortality rates. Therefore, this study further evaluated the differences in sCP levels among patients exhibiting various clinical manifestations. Results demonstrated significantly higher sCP levels in patients presenting renal injury, hepatic injury, and neurological symptoms compared to those without these complications (Figures 6A–C). However, no significant differences in sCP levels were observed between patients with or without hypertension and diabetes (Figures 6D,E).

## 4 Discussion

CP exhibits multiple regulatory functions in immune defense, including neutrophil chemotaxis and chelation of various divalent metal ions (Zygiel and Nolan, 2018). In recent years, CP has been extensively employed as a biomarker not only in IBD, but it has also increasingly become a research focus in various inflammatory disorders. fCP, a widely validated non-invasive biomarker for IBD, effectively assesses intestinal inflammatory activity (Mosli et al., 2015; Kyle et al., 2021). fCP levels significantly correlate with clinical and endoscopic disease activity, aiding in differentiating inflammatory from non-inflammatory bowel disorders. Its excellent stability, reproducibility, and low assay cost render it a valuable diagnostic and monitoring tool for IBD.

An increasing number of studies have highlighted sCP as a potential biomarker for various inflammatory diseases, in addition to fCP (Schoepfer et al., 2008; Dai et al., 2015; Kalla et al., 2016; Inciarte-Mundo et al., 2022). In rheumatoid arthritis, sCP promotes chemotaxis, migration, and regulatory functions of neutrophils and macrophages. Multiple studies suggest that sCP stratifies disease activity more precisely than conventional markers such as CRP and ESR, underscoring its substantial biomarker potential. Additionally, sCP levels significantly increase in SLE (Manfredi et al., 2023), ankylosing spondylitis (Oktayoglu et al., 2014), periodontitis (Lira-Junior et al., 2017), and various malignancies [e.g., myelodysplastic syndromes (Giudice et al., 2019)], closely correlating with disease activity and patient quality of life. Notably, sCP has demonstrated clinical relevance in various viral infections, reflecting systemic inflammation and disease severity (Mahler et al., 2021). Our current study observed notably elevated sCP levels in SFTS patients, positively correlated with viral load, multiple clinical inflammatory markers (e.g., CRP, ALT), and serum cytokines (e.g., TNF-α, IL-6). This suggests that sCP may serve as an inflammatory marker in SFTS. Although sCP is not a specific biomarker for SFTS, the focus of our study is not on its diagnostic value in SFTS but on its use as an indicator of inflammation severity and disease progression. sCP is mainly released by neutrophils and reflects neutrophil activation and systemic inflammatory status. We found that sCP levels closely correlated with disease severity in SFTS patients, being significantly elevated in those with severe disease and in non-survivors. This elevation may be related to virus-induced cytokine storm and immune dysregulation, suggesting a potential mechanistic role in disease progression. However, ROC curve analysis revealed only moderate predictive power of sCP alone for disease severity and mortality. Therefore, combining sCP with other inflammatory or clinical markers may enhance prognostic accuracy in SFTS. While fCP is widely used as a marker of intestinal inflammation in IBD, sCP levels in IBD patients are generally not markedly elevated unless systemic inflammation is present. Therefore, by combining sCP measurement with clinical presentation and viral testing, SFTS and IBD cases can be effectively distinguished especially in SFTS patients without obvious gastrointestinal symptoms. In addition, we excluded participants with active IBD in this study to avoid confounding bias.

Neutrophils and monocytes/macrophages constitute the primary cellular sources of CP (Chen et al., 2023). The release of CP occurs through a non-classical secretion pathway, which does not rely on the endoplasmic reticulum-Golgi apparatus. Upon inflammatory stimulation, neutrophils can release calprotectin into the extracellular space through degranulation, cellular stress, or even cell death (such as necrosis or NETosis). The protein then enters the bloodstream, forming detectable levels of sCP (Vogl et al., 2012; Foell et al., 2009). In the present study, significant correlations emerged between sCP levels and peripheral blood leukocyte subsets, notably leukocyte counts, lymphocytes, monocytes, and basophils. These correlations suggest that, during viral infections, CP sources may be closely linked to activation or proliferation states of these immune cells. Although lymphocytes do not directly produce CP, they may indirectly promote CP generation by cytokine-mediated stimulation of monocytes. Basophils, key players in allergic and inflammatory responses, may indirectly affect CP production by releasing histamine and other inflammatory mediators. Our observation of no significant correlation between sCP and neutrophil counts may be attributable to local accumulation of neutrophil-derived CP at inflammatory sites (e.g., mucosal tissues). Thus, the increased sCP levels in SFTS patients likely originate predominantly from monocyte/macrophage populations.

Furthermore, this study revealed significantly elevated sCP levels in SFTS patients compared to healthy controls. Patients with severe disease exhibited higher sCP levels than those with mild disease, and non-survivors showed higher levels than survivors. This finding aligns with results from other inflammatory diseases, such as COVID-19 and SLE, further substantiating the utility of sCP as a severity marker. Cytokine storm represents a critical mechanism underlying SFTS deterioration. Our data demonstrated significant positive correlations between sCP and pro-inflammatory cytokines, including TNF-α, IL-6, IL-8, and IL-10, suggesting a close association between sCP and cytokine storm. Cytokine storm has been recognized as a critical mechanism driving disease progression in various viral infections, involving excessive release of pro-inflammatory cytokines such as IL-6 and TNF-α (Khaiboullina et al., 2017; Montazersaheb et al., 2022). Similarly, sepsis involves massive pro-inflammatory cytokine release (e.g., TNF-α, IL-1β, IL-6), triggering systemic inflammatory responses and tissue injury (Chousterman et al., 2017). Our results align with these mechanisms, as the significant correlations between sCP and various cytokines indicate its pivotal role in the inflammatory cascade, indirectly reflecting disease severity.

Regarding laboratory parameters, we observed significant positive correlations between sCP and CRP, AST, LDH, TT, and D-dimer. Among these markers, AST, LDH, and D-dimer have been reported as important laboratory markers reflecting inflammation, tissue damage, and coagulation dysfunction in various infectious diseases (Battaglini et al., 2022; Xia et al., 2024). The significant association between sCP and these markers in our study further suggests its clinical relevance in SFTS, reflecting inflammation severity, tissue injury, and coagulation dysfunction.

Given sCP’s strong correlations with various inflammatory indices, we hypothesize a potential role of sCP in the pathogenesis of SFTS. Previous studies indicated that, in chronic inflammation (e.g., IBD), intestinal epithelial cells express CP upon inflammatory stimulation, recruiting immune cells and releasing pro-inflammatory mediators, thereby establishing a positive feedback loop (Jukic et al., 2021). Experimental evidence also demonstrates that sCP enhances immune cell chemotaxis and migration and promotes inflammatory mediator release, acting as an arachidonic acid-binding protein to facilitate the synthesis of lipid mediators, such as leukotrienes, further amplifying inflammation (Wang et al., 2018). Given our observation of positive correlations between sCP and inflammatory markers, we propose a similar positive feedback loop in SFTS: inflammatory stimuli induce abundant CP production and release, which, in turn, exacerbate inflammation, creating a vicious cycle. However, additional experimental studies are necessary to confirm this hypothesis.

Despite the evidence provided herein for a correlation between sCP and SFTS severity, the study has limitations. The relatively small sample size necessitates further validation through larger-scale, multicenter studies. Furthermore, although we observed that sCP might participate in inflammation regulation in SFTS through activation of lymphocytes and stimulation of cytokine release, its precise mechanisms in SFTS pathogenesis remain unclear and require further investigation through both basic and clinical studies.

In summary, this study is the first to identify clinical associations of sCP with SFTS. Our findings reveal significantly elevated sCP levels in SFTS patients, positively correlated with viral load, thrombocytopenia severity, and multiple pro-inflammatory cytokines (TNF-α, IL-6, IL-8, IL-10). Additionally, sCP correlated with clinical indicators such as CRP, AST, LDH, and D-dimer, underscoring its potential as a comprehensive biomarker for inflammatory storms, tissue injury, and coagulation dysfunction. The correlation of sCP levels with complications like renal injury and neurological symptoms further highlights its clinical utility as an early warning indicator. Collectively, our results suggest that sCP holds promise as a valuable diagnostic and prognostic biomarker in SFTS.

## Data Availability

The raw data supporting the conclusions of this article will be made available by the authors, without undue reservation.
