# Supplementary material for: Serum calprotectin correlates with severity of severe fever with thrombocytopenia syndrome
Source: Front Microbiol. 2025 Jun 27;16:1604243. doi: 10.3389/fmicb.2025.1604243 (PMC12246975; doi:10.3389/fmicb.2025.1604243)
Supplement: Supplementary file 1 [file Data_Sheet_1.DOCX]

Serum calprotectin correlates with severity of severe fever with thrombocytopenia syndrome

**Shijie Cai^1,^** **^†^, Jiahua Zhu^1,^** **^†^, Zhiye Xu^2^, Wenqin Chen^2^, Yue Tao^2, *^, Taihong Huang^2, *^, Sen Wang^1, 2, *^**

^1^ Department of Clinical Laboratory Medicine, Nanjing Drum Tower Hospital Clinical College of Nanjing University of Chinese Medicine, Nanjing 210000, China.

^2^ Department of Clinical Laboratory Medicine, The Affiliated Drum Tower Hospital of Nanjing University Medical School, Nanjing, China.

*** Correspondence:**Sen Wang, Taihong Huang, Yue Tao

njwangsen@163.com; 1262117241@qq.com; peachmoon@163.com

Shijie Cai ^1, †^, Jiahua Zhu ^1, †^， †These authors contributed equally to this work and share first authorship

Sen Wang^1, 2, *^, Taihong Huang^2, *^, Yue Tao^2, *^, ^*^These authors contributed equally to this work and share corresponding authorship

**
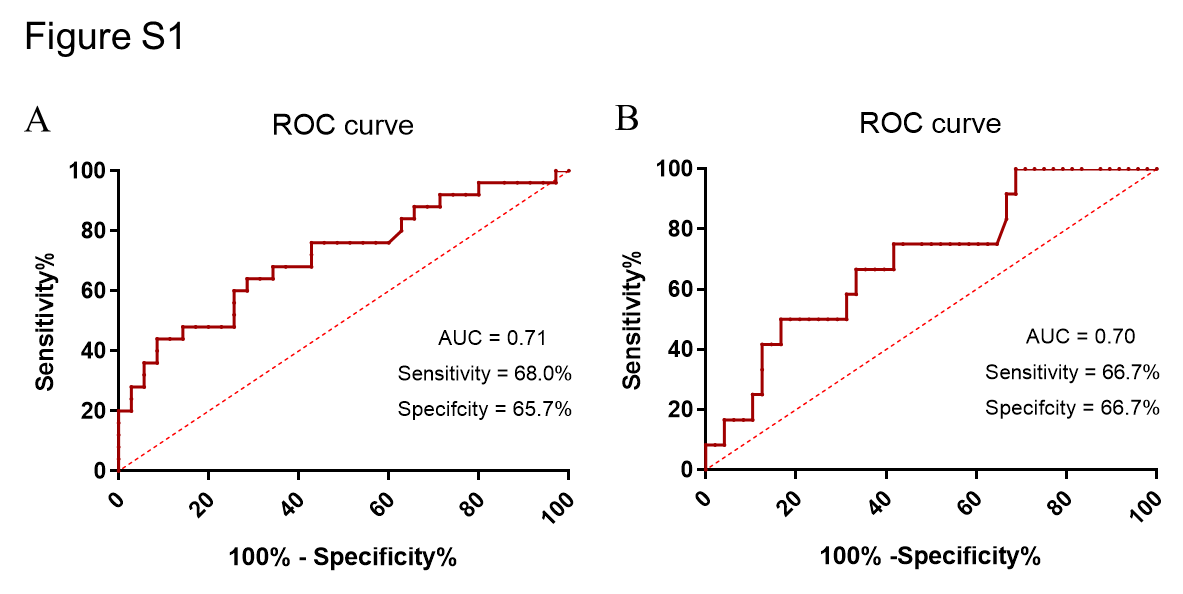
**

Figure S1. ROC curve analysis of sCP levels for distinguishing disease severity and predicting mortality in SFTS patients. The AUC for differentiating severe from mild cases was 0.71, with a sensitivity of 68.0% and specificity of 65.7% (A). For predicting mortality, the AUC was 0.70, with both sensitivity and specificity of 66.7% (B).


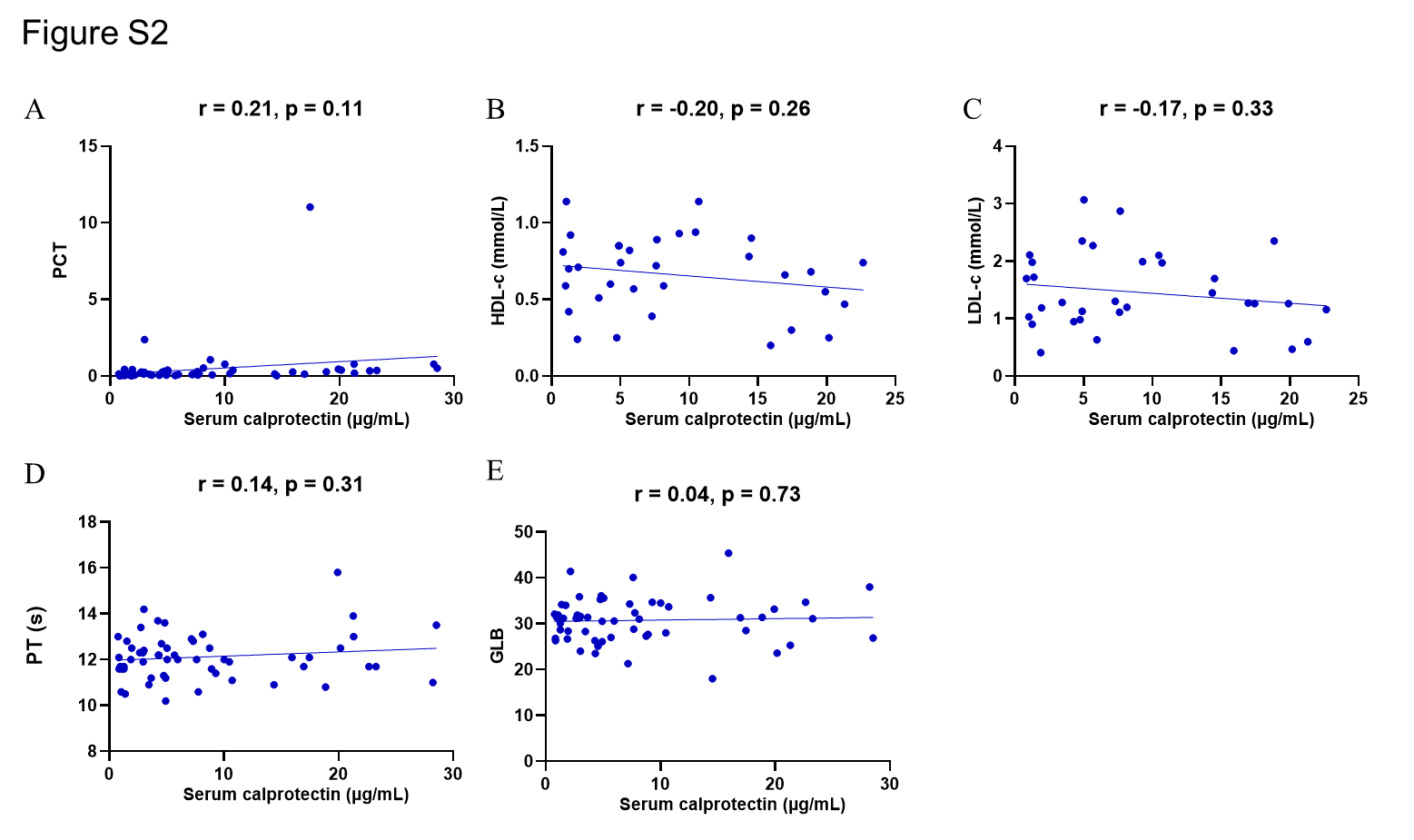


Figure S2. Correlation of sCP with clinical parameters. sCP levels in SFTS patients were analyzed for correlation with PCT, HDL-c, LDL-c, PT and GLB, respectively (A-E).
